# The Effects of Kinesio Taping^®^ on Muscle Interplay within the Lumbo–Pelvic–Hip Complex: A Randomized Placebo-Controlled Trial

**DOI:** 10.3390/sports11030070

**Published:** 2023-03-17

**Authors:** Dalibor Kiseljak, Vladimir Medved

**Affiliations:** 1 Department of Physiotherapy, University of Applied Health Sciences, 10000 Zagreb, Croatia; 2 Faculty of Kinesiology, University of Zagreb, 10000 Zagreb, Croatia

**Keywords:** kinesio taping, intermuscular coordination, prone hip extension, surface electromyography, onset

## Abstract

Coordination of muscle activity is determined by the recruitment order of agonists and synergists that results from their onset times. Motor recruitment deficits are possible. This study examined the acute and prolonged effects of three different techniques of the kinesio taping method in optimizing the intermuscular coordination within the lumbo–pelvic–hip complex. The sample consisted of 56 healthy participants of both genders, randomly divided into equal groups by kinesio taping muscle facilitation, muscle inhibition and functional correction technique, and placebo kinesio taping condition. The onsets of the ipsilateral and contralateral erector spinae muscles, in relation to the semitendinosus muscle of the tested leg, were measured using the surface electromyography, during the active performance of the prone hip extension test. Time span was also determined. Measurements were performed at baseline, 60 min, and 48 h post-intervention. For the control group, we did not find statistically significant differences in the onset between the measurement points (*p* > 0.05), while in the experimental groups, there was a significant delay in the onset of the contralateral erector spinae (*p* < 0.001) in the second and third measurement points. These results indicate that the kinesio taping method can optimize the intermuscular coordination, with the potential for primary injury prevention.

## 1. Introduction

The lumbo–pelvic–hip complex is a paradigmatic entity in the analysis of human posture, with structural and functional connections of the trunk and lower extremities [1]. Optimal recruitment patterns of synergistic muscles with an appropriate order are considered important for efficient lumbar spine function [2]. Changes in muscle activation patterns are manifested by shortened or delayed onset times. Changes in onset can result in changes in the recruitment order to perform a specific motor task. Thus, for example, hip extension can be compensatory initiated and dominantly performed by trunk extensors.

Prone hip extension test is used to examine the function of the lumbo–pelvic–hip complex [3], through the assessment of the motor pattern of the lumbo–pelvic–hip complex, i.e., intermuscular coordination in the background of segmental hip extension with the lower leg extended [4].

Kinesio taping is a therapeutic method developed in Japan at the beginning of the last quarter of the twentieth century. It includes eight different techniques using a self-adhesive elastic tape that, by imitating the elastic properties of the skin, replicates the tactile stimulus of the therapist’s hands placed on the skin [5].

Kinesio taping is used in the prevention and rehabilitation of various conditions related to sports and recreational, as well as professional, activities in the general population. It can lead to a reduction in pain [5], the most common symptom resulting from muscle and connective tissue dysfunction with repercussions on joints, whilst not preventing or limiting movement. Kinesio taping provides active support to the musculoskeletal system, through the improvement of impaired proprioception [6] and through the optimization of blood and lymphatic circulation [7], with the aim of faster tissue recovery [8,9] and the normalization of functions [6,10]. There is a possibility of symptomatic, as well as causal, therapeutic effects of the kinesio taping method. Therefore, in addition to the analgesic effect on tissues and decongestion, Kase et al. [5] emphasized the normalization of muscle function and the correction of joint malalignment as the physiological and biomechanical basis of kinesio taping, aspects that can contribute to the optimization of posture and movement, even among asymptomatic individuals.

The mechanism of the kinesio taping functional correction technique results from the specific way of placing the kinesio tape, with the application of more than three-quarters of the maximum tension (75+%). The remaining elasticity through the spring effect assists the movement (e.g., hip flexion), at the same time limiting the antagonistic movement (e.g., hip extension) through the principle of preload, where the proprioceptors send the information of the repositioned end of ROM as normal, even before the full ROM [5]. Therefore, kinesio taping functional correction technique can be used to increase mechanoreceptor stimulation to assist or limit movement at a particular joint or kinetic chain. Contrary to kinesio taping techniques for facilitating or inhibiting muscle function, functional correction affects the functional movement pattern, not the tissues. The application procedure is more complex, compared to all other kinesio taping techniques [5], from positioning the patient, controlling the percentage of tension, to anticipating the change in segmental posture of the joint, from initial to final. These factors could be the reason that functional correction is the least studied kinesio taping technique. To the best of our knowledge, we found that kinesio taping functional correction technique was used in only one study [11], where it was an accompanying technique to kinesio taping muscle facilitation, without differentiating the effects of the two techniques.

Although the lumbar region has been extensively studied, in the context of kinesio taping [12,13,14,15], this is not the case for the hip region. Numerous studies have been conducted on the effectiveness of kinesio taping in various injuries and disorders of the neuro-musculoskeletal system. There is much less research on the effect of kinesio taping in healthy people. Lumbroso et al. [16] considered that, if the application of kinesio taping can affect the strength or flexibility of a healthy muscle, it can be used in cases of muscle imbalance, which is important in the treatment and prevention of musculoskeletal pathology.

In the available literature, we did not find any research on the influence of kinesio taping on the intermuscular coordination of the lumbo–pelvic–hip complex. Comparing the muscle interplay in prone hip extension of asymptomatic and symptomatic subjects, Bruno et al. [17] emphasized the need for future research on the influence of exercise or some other intervention modality on the restitution of “normal” muscle activation patterns. Considering the abovementioned, we decided to investigate the effects of the kinesio taping method through the analysis of its three different techniques. We were guided by the fact that the kinesio taping functional correction technique has not been scientifically tested so far, while muscular techniques have, although with some contradictory conclusions.

The aim of this study was to electromyographically examine the acute and prolonged effects of three different kinesio taping techniques on the intermuscular coordination in prone hip extension. We hypothesized that muscle interplay will be significantly improved by applying kinesio taping, in the direction of better post-intervention recruitment results, especially after 48 h, and that the interactions between the group and the measurement point will be statistically significant.

## 2. Materials and Methods

### 2.1. Study Design

A single-blind, randomized, placebo-controlled trial was conducted with healthy participants. Using the block randomization procedure (R, version 3.6.2), the participants were divided into five groups. The slips with the codes were sealed by an independent expert in opaque envelopes, which were opened by the participants. In each case, the dominant leg was tested (and treated). In the first group, we applied the kinesio taping functional correction technique (FC). Kinesio taping for the correction of muscle function was applied in the second (with proximal anchor placement (PDF principle—proximal to distal = facilitation)) and third (with distal anchor placement (DPI principle—distal to proximal = inhibition)) groups. The fourth group received placebo kinesio taping (PKT). The first three groups were experimental, and the fourth was control.

The research was conducted in the Faculty’s Physiology Laboratory. All measurements and interventions were performed by a certified kinesio taping instructor. The main measurements were performed at three time-points: at baseline (i.e., not having received the intervention), 60 min, and 48 h post-intervention. All measurements, at all time points, were made approximately at the same time of day, in the afternoon. The participants had to be rested, i.e., a minimum of 12 h without intense physical activity before the initial tests, but also for the next 48 h until the final measurements. The Faculty’s Science and Ethics Committee gave approval for the research (decision no: 19/17). Prior to the examination, the participants signed the informed consent form.

### 2.2. Participants

The sample consisted of 56 students (37 women and 19 men), with the average age of 23.1 ± 4 years, ranging from 19 to 40 years. Their average height was 172.9 ± 8.9 cm, weight 71.2 ± 15 kg, with BMI 23.7 ± 4.1 kg/m^2^.

The required sample size was calculated using the computer program G*Power 3.1. Forty-four participants (11 per group) were needed to achieve statistical power of 0.9 and alpha error level of 0.05. We included about 25% larger sample in the study.

The inclusion criteria were: ages 18–40, healthy, no previous experience with kinesio taping.

The exclusion criteria were: lumbo–pelvic–hip complex and lower extremity injuries in the last six months, neuromuscular disorders, structural scoliosis, pain when performing the test, skin diseases, allergic reaction to Kinesio material, and pregnancy in the two previous years.

### 2.3. Procedures

**Figure 1:**
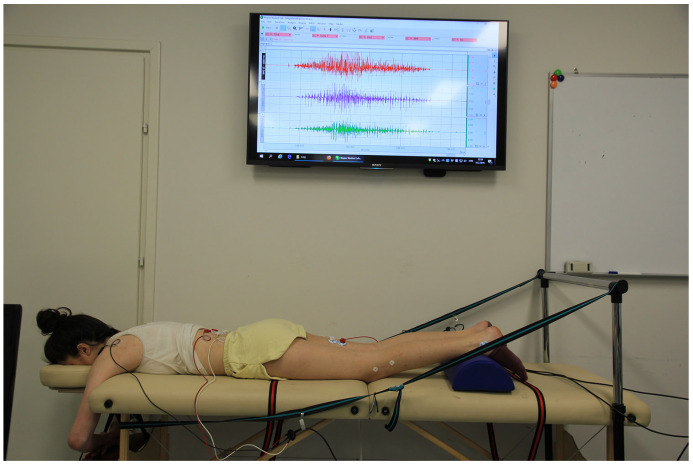
Starting position of the participant for the Prone Hip Extension test.

The subjects’ height and weight were measured, along with simultaneously the dominant leg was detected (determined as the leg with which the subject stepped onto the measuring station, Seca GmbH & Co. KG., Hamburg, Germany). This method of determining the dominant leg is complementary to Lin et al. [18]. The electromyographic features of lumbo–pelvic–hip complex intermuscular coordination were measured during active performance of prone hip extension on the examination table, according to a recently described testing procedure [19], (see also Figure 1), using a 4-channel sEMG device (BIOPAC MP35, Biopac Systems, Inc., Goleta, CA, USA). Signal acquisition was performed at the sampling rate of 1000 Hz. Assessment of intermuscular coordination was based on onset times and time span of activation of three muscles: ipsilateral semitendinosus (ST), erector spinae (ESI), and contralateral erector spinae (ESC).

Skin preparation for electrode placement included body hair trimming (if deemed necessary) and abrading the skin surface using the rough sponges (ELPAD 2.5 cm × 5 cm). Disposable, self-adhesive Ag/AgCl snap electrodes (BIOPAC EL503) were placed in bipolar parallel to the muscle fibers and with a 2 cm distance from the center to the center of each other, on defined muscle points, according to SENIAM recommendations (http://www.seniam.org, accessed on 1 January 2023). Reference electrodes were placed on the acromial processes and on the lateral malleolus of the tested leg. The electrode placement zones did not conflict with the application of the therapeutic intervention or the performance of the test movement.

We wanted to determine realistic indicators of the activities of daily living; therefore, no warm-up was performed. Before starting the data acquisition, the prone hip extension test was explained and demonstrated to the participants in detail, and they made several test attempts. Then, the recording of 20 repetitions began.

To ensure movement at a natural speed, with a proper rhythm (5 s each for the activation and relaxation phases), we used verbal guidance with repetition counting. Visual feedback by controlling the electromyogram in real time via the screen was available only to the examiner (see Figure 1). EMG signals were subject to analog-to-digital conversion. The data were stored in a personal computer and presented in the form of graphs suitable for further processing.

### 2.4. Data Reduction

EMG signal processing was performed using the BIOPAC Student Lab Software v. 4.1.3 PRO. Raw electromyography signals were band-pass filtered (28–500 Hz) and full-wave rectified. Artifacts during the first and last prone hip extension attempts were observed by visual inspection in many participants. Therefore, we included the signals obtained at the central 18 repetitions in the analysis.

The main analysis of the EMG records was the determination of the onset time for each repetition of each muscle, based on the detected peak value of muscle activity. A muscle is considered activated when the rectified signal exceeds 10% of the rectified peak amplitude for that muscle during prone hip extension [20]. The variables peak value and onset time were identified by software, and we confirmed them by visual inspection of all EMG records.

The data were exported to Microsoft Excel, where a comparison of the recruitment onsets of three muscle groups was made. Prior to the comparison, the onsets were normalized using the method according to onset_ST_ = 0 ms [21].

In this way, the variable normalized onset (NONSET) was formed for all muscles, for each attempt of each subject. The pre-activation of ESC/ESI, in relation to NONSET_ST_, received a negative sign. NONSET values were averaged (the average of the 18 repetitions) to form the main variable for quantitative analysis—average NONSET (ANONSET). Based on the recruitment onsets, we also determined the time span, between the first (any) and the last (any) activated muscle for each repetition. Then, based on the average of the 18 repetitions we calculated the average value for each subject, for each point of measurement, forming the variable average time span (ATS).

### 2.5. Tape Applications

In each of the applications, the same material (Kinesio Tex Gold FingerPrint Tape, Kinesio Holding Company, Albuquerque, NM, USA, 5 cm wide, black) was used, so that the type of tape [22] would not influence the results. The kinesio taping application was preceded by cleaning the skin with alcohol, trimming the hairs (if deemed necessary), and placing the subject in a position specific to each intervention.

#### 2.5.1. Functional Correction Technique Application

**Figure 2:**
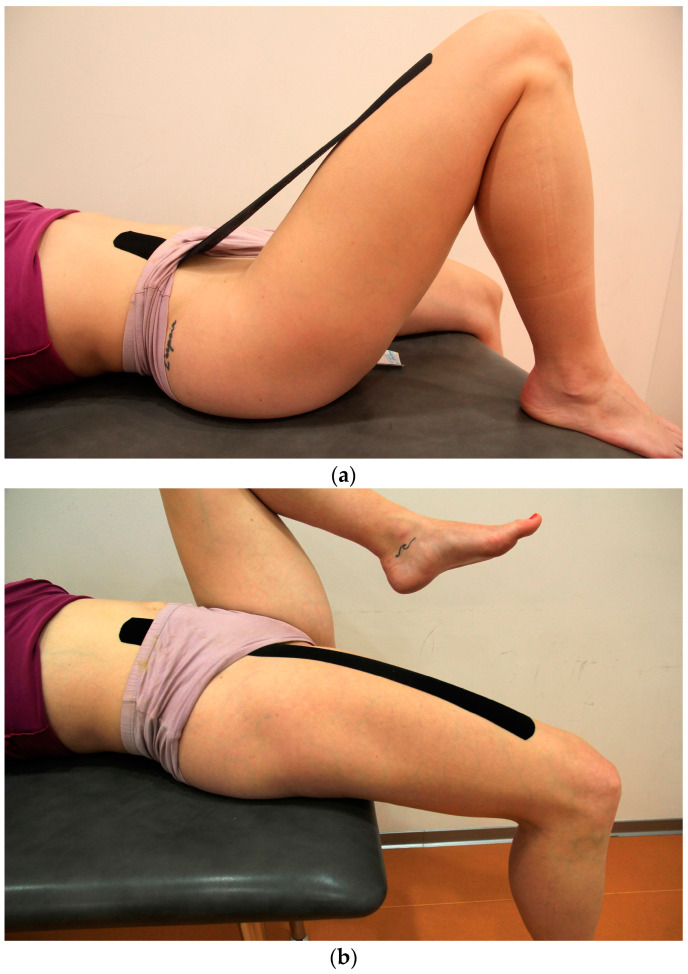
(**a**) Application of the Kinesio Taping Functional Correction technique—first step. (**b**) Application of the Kinesio Taping Functional Correction technique—final step.

The participants assumed a supinated position. Kinesio tape 40 cm long was placed in the anterior pelvic region with the first 5 cm without tension. The participants flexed the upper leg up to 45°, and the therapeutic zone was stretched to 50% by the straight line, and the end without tension was placed on the anterior part of the thigh (see Figure 2a). This was followed by a procedure [5] of placing the therapeutic zone on the skin, whereby the upper leg was maximally extended (see Figure 2b), and the tape that was stretched in the air was adhered to the skin, with a final tension in the therapeutic zone between 75 and 100% (however, in order to make wearing during the next two days more comfortable, we avoided going to the maximal final stretch, and hence it was between 80 and 90%).

#### 2.5.2. PDF Application

The participants were in the supinated position, and the anchor (the first 5 cm) of a 50 cm long Kinesio tape was placed, without tension, on the proximal attachments of the hip flexors. Then, the therapeutic zone of the tape with 25% tension was placed on the stretched muscles, and the end, without tension, was adhered to the anterior distal part of the upper leg in the same position.

#### 2.5.3. DPI Application

Kinesio tape of identical shape and length as in the PDF group was applied with the same tension, but according to the reverse principle, the anchor was first placed distally in a neutral position.

#### 2.5.4. Placebo Kinesio Taping Application

**Figure 3:**
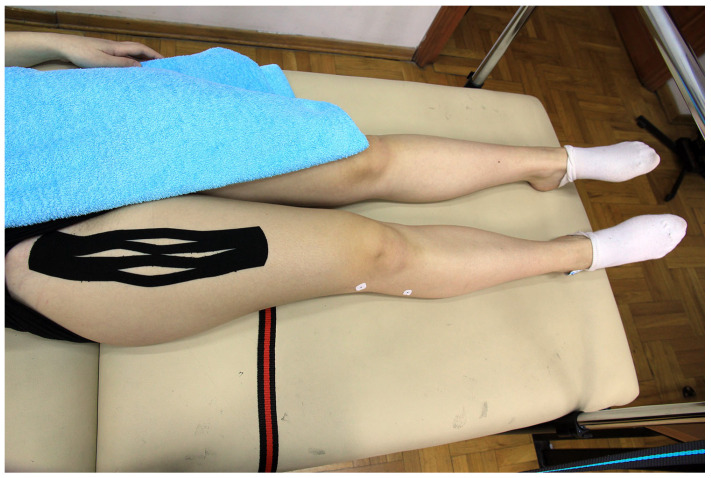
Placebo Kinesio Taping application.

Kinesio tape 30 cm long was prepared as a basket weave cut [5], but recoil was neutralized in the therapeutic zone (by stretching the tape to 100% and returning to 0% tension), and the entire tape, without any tension, was placed on the anterior thigh which was in a neutral position (see Figure 3).

### 2.6. Statistical Analyses

Data were analyzed using the SPSS software package, version 18 (SPSS Inc., Chicago, IL, USA). Basic descriptive indicators were calculated, and the groups were compared according to their characteristics using the χ^2^ test and one-way ANOVA. The main analysis was performed using a 3 × 4 mixed-model ANOVA, with intervention type as an independent factor and time-point as a dependent factor. For the independent component of the analysis, Levene’s test was used to test the assumption of variance homogeneity among groups. For the dependent component of the analysis, sphericity was assessed by Mauchly’s test. In violation of Mauchly’s test, the Greenhouse–Geisser correction was used.

With the assumption of sphericity and homogeneity of variances, the main analysis—testing the effects (main effects of group and time-point, and their interaction)—was performed. According to Cohen [23], eta squared (η^2^) was used to classify the effects (i.e., small (0.01–0.05), medium (0.06–0.13), and large (>0.14)). In cases where significant main effects were determined, individual comparisons between variables were performed using the Fischer’s post-hoc LSD test. The same test was used for the analysis of simple effects, in case of a significant interaction. The level of statistical significance was set at *p* ≤ 0.05.

## 3. Results

The analysis showed that the randomized groups are homogeneous, regarding the gender and age, body height, body weight, and body mass index.

The main descriptive results of electromyography for the average normalized onset times (in milliseconds) of the contralateral (ANONSET_ESC_) and ipsilateral (ANONSET_ESI_) erector spinae muscles, in relation to the normalized onset time of the semitendinosus muscle (NONSET_ST_ = 0 ms) during the performance of prone hip extension, are presented in Table 1 and Table 2. Table 3 shows the descriptive parameters of average time span.

The main findings of this research regarding the hypotheses are presented in Table 4.

For the ANONSET_ESC_ and ANONSET_ESI_ variables, post-hoc testing revealed significant differences (*p* < 0.001) between the first and second and first and third time-points, in the direction of higher post-intervention values, while the difference between the second and third time-points is not statistically significant (*p*_ESC_ = 0.118; *p*_ESI_ = 0.77).

**Figure 4:**
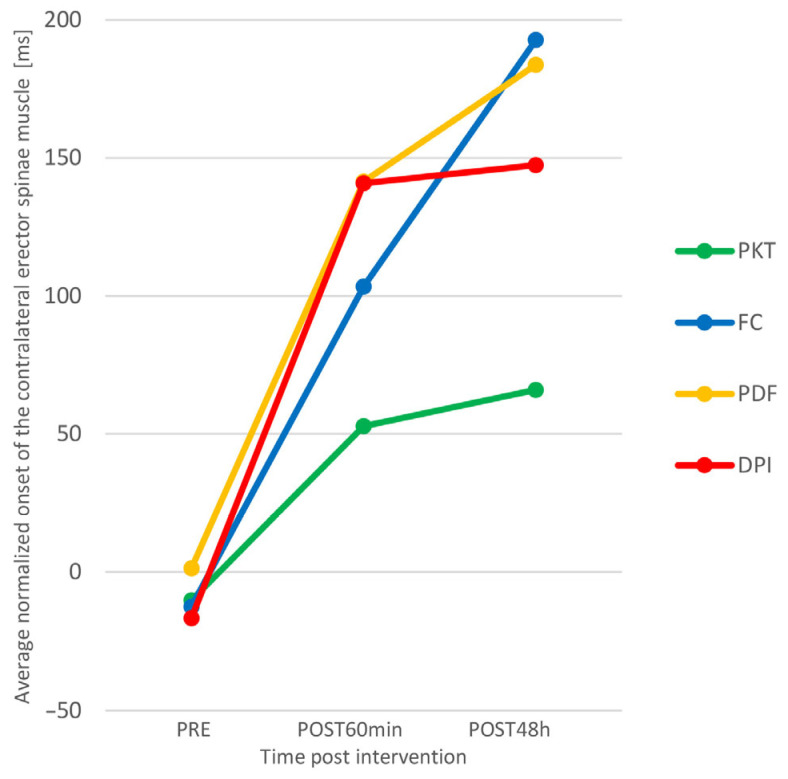
Average normalized onset of the contralateral erector spinae muscle (ANONSET_ESC_) interaction plot for 4 groups (PKT—Placebo Kinesio Taping; FC—Functional Correction; PDF—proximal to distal = facilitation; DPI—distal to proximal = inhibition).

Figure 4 suggests that there was a greater increase in ANONSET_ESC_ in the experimental groups than in the control group. We checked this by analyzing simple effects.

It was shown that, in the control group, there is no significant difference between individual time-points, while in the FC group, the differences between all time-points are statistically significant in the direction of better results (higher ANONSET_ESC_), and in the PDF and DPI groups, there are significant differences between the first and second and the first and the third point, also towards better results. Functional correction proved to be the most effective intervention technique, since there was a significant increase in ANONSET_ESC_ (both acutely and prolonged) in the group where Kinesio tape was applied in this way.

The link between the NONSET_ESC_ and NONSET_ESI_ results is the recruitment order variable. As we examined, for three muscles, there were six possible recruitment orders. By comparing the mean values of NONSET_ESC_ and NONSET_ESI_ of all subjects between different measurement points, we can interpret that at baseline the dominant recruitment order of all subjects was ESI-ST-ESC, while at the second and third measurement points, it was ST-ESI-ESC, which is a typical [19] muscle recruitment pattern for prone hip extension. On average, the intervention had a positive effect on recruitment order, i.e., it led to an improvement in intermuscular coordination.

## 4. Discussion

The main finding of the research is that the application of kinesio taping to the hip flexors can improve the intermuscular coordination of the lumbo–pelvic–hip complex in healthy individuals. Our findings show that, in the experimental groups, there are significant acute and prolonged post-interventional changes in the myoelectric activity of agonists and synergists of hip extension. Kinesio taping led to a significant increase in ONSET_ESC_ and ONSET_ESI_, followed by the improvement in recruitment order, in such a way that semitendinosus muscle assumed an agonistic position in the motor pattern of prone hip extension.

The electromyographic indicators of the control group did not change significantly over time, while in the experimental groups, we found significant positive changes. In addition to the FC group, where we found statistically significant differences between all measurement points, in the PDF and DPI groups, significant differences were found, also in the direction of optimizing intermuscular coordination, between the first and second and first and third points. Our findings correspond to the results of Słupik et al. [24], who found an increase in the electromyographic activity of the vastus medialis muscle 24 h after the application of Kinesio tape, with the effect being maintained for another 48 h. We also see a connection with studies [25,26,27] that found an increase in the myoelectric activity of the gastrocnemius muscle after the application of kinesio taping. The research of Martínez-Gramage et al. [28] was most closely related to our experiment, and it showed that the DPI technique has a significant prolonged effect (72 h after the intervention), in terms of increasing the onset of the lateral gastrocnemius muscle during walking. This supports the theory of kinesio taping as a method that leads to a change in muscle activity. Our results contradict those of Fu et al. [29] on the absence of an effect of kinesio taping on the myoelectric activity of the rectus femoris muscle, which was also concluded by Lins et al. [30,31], who indicated that Kinesio tape applied to the rectus femoris, vastus lateralis, and vastus medialis muscles does not change the lower limb neuromuscular function of healthy people, either acutely [30] or prolonged [31]. Briem et al. [32] found no significant differences for the average and maximum EMG activity of the fibularis longus muscle during ankle inversion perturbations in healthy athletes, thus disputing the benefit of kinesio taping for the primary prevention of ankle sprains. The possible reason for the absence of the effect lies in the fact that the researchers made holes for the EMG electrodes on the Kinesio tape, which unavoidably impaired the effect of the material, and apparently recoil, as a key mechanism [5] for changing muscle function was completely absent. In several studies [29,31,33,34], we found a major drawback in the way kinesio tapes are placed—starting from the belly of the muscle about 10 cm distal to the proximal attachment, which means that there was no effect on the entire muscle. Covering the attachments is an important link in the entire proprioceptive and neuromuscular essence of kinesio taping [5]. We believe that the imprecise placement can be a sufficient reason for the absence of the kinesio taping effect. A glaring example is the study [35] on the direct effects of kinesio taping facilitation and inhibition techniques on the EMG activity of the gastrocnemius muscle of healthy subjects. The credibility of the finding that these techniques have no significant effects is quite questionable, considering that the Kinesio tape was placed far from the muscle attachments, with approximately 50% of the anatomical length left uncovered. We also find a potential problem in the use of 50% tape tension, which apparently could have created a positional hold, instead of a recoil effect. Positional hold should never be applied to the muscles [5] because it does not provide decompression and mobilization, but has the opposite effect. To avoid such a mistake, Kase et al. [5] recommended 15–25% tension for inhibition and 15–35% for facilitation.

We found significant changes in intermuscular coordination, both acute and prolonged. Here, we find a difference, compared to Takasaki et al. [3], who detected no significant changes in the onset neither for ESC nor for ESI, following the intervention by applying manual pressure on the pelvis of asymptomatic subjects.

For the time span variable, none of the research hypotheses were confirmed. For the time factor, apart from finding no statistically significant differences, there is no noticeable trend. We interpret this as an indicator of the motor pattern variability of normal movement described by Latash et al. [36], in the context of the theory of motor redundancy of a system with multiple elements and a constant variable value. Therefore, in the background of the movement pattern, where the time span provides the framework, there may be different motor patterns that are reflected through different recruitment order, which, similar to the onset, is subject to changes due to the intervention. Intermuscular coordination showed variability of onset, due to different interventions; however, the time span, as a frame, remained constant with average values of 0.397 s in the first measurement point, 0.396 s in the second, and 0.385 s in the third, being a very stable variable, with no statistically significant differences for any observed effect. Analyzing muscle interplay at prone hip extension, Lehman et al. [21] obtained an average time span of 0.37 s, and Bullock-Saxton et al. [37] obtained 0.306 s. The latter included a group of participants with ankle distortion in the study, whose average score of 0.527 s was significantly higher. Therefore, the time span is suggested as an indicator of deviation from the normal motor pattern.

As far as we know, this is the first study that investigated the influence of the application of kinesio taping on the myoelectric activity of the lumbo–pelvic–hip complex when performing prone hip extension in young asymptomatic subjects. We did not find any study that, by analyzing the activation patterns, evaluated the effects of the kinesio taping method on lumbo–pelvic–hip complex intermuscular coordination of healthy or symptomatic subjects. Furthermore, to the best of our knowledge, this is the first attempt to investigate the kinesio taping functional correction technique. In the available literature, we found only one study [11] where the mentioned technique was used, although not separately, but as part of a comprehensive kinesio taping intervention. Therefore, there are no published results that compare functional correction to other kinesio taping techniques or some other therapeutic interventions, which is astonishing, considering the widespread usage of the kinesio taping method in clinical and sports practice, which has been followed by a considerable number of studies in recent years.

According to Drouin et al. [38], the evidence that would support the use of kinesio taping in the function of improving sports performance by optimizing strength, proprioception, or ROM in healthy individuals is insufficient. On the other hand, they state that there is no argument that kinesio taping generates any negative effect. They also point out that there is evidence that kinesio taping can improve bioelectrical activity prolonged (24, and 72 h after application), but not acutely in the period from 0 to 45 min after application. We believe that, with our research, we have supplemented the results of the aforementioned review, mainly through the finding of a positive change in myoelectric activity 60 min after application. Most published research on the effects of kinesio taping in healthy individuals analyzed only acute effects [11,14,15,27,29,30,31,34], while we repeated all measurements after 48 h of wearing the Kinesio tape, which was also performed by few authors [16,24,26,31,39]. Only some of the studies included a placebo intervention [11,14,31,33,34,40,41,42]. However, in many of them [14,31,34,40,41,42], due to the simple placement of a piece of kinesio tape onto the skin without pre-stretching, placebo kinesio taping inadvertently became an active intervention factor, which, in the kinesio taping method, is used within the epidermis–dermis–fascia medical taping application in patients highly sensitive to external stimuli [43]. Therefore, such an application of kinesio tape cannot be considered a placebo, but rather an active intervention that provides a different level of stimulation [39]. In our effort to conduct a quality placebo-controlled trial, the experience and knowledge of the principal investigator, a licensed kinesio taping practitioner and instructor, were highly beneficial in the preparation and application of the placebo kinesio tape.

### 4.1. Limitations and Future Research Perspectives

Our study has some limitations. The sample was convenience, and the examiner was not blinded. Prolonged effects after 48 h were examined, but not those after 72 and/or 96 h. No follow-up testing was performed after the removal of kinesio tape, which would be interesting to analyze and compare with the results of studies [24,31,44] that also investigated this. It would be desirable to check the influence of kinesio taping on muscle interplay during closed kinetic chain movements and in combined activities, such as walking and some sport-specific movements, as well as in symptomatic individuals.

### 4.2. Practical Application of the Study

**Figure 5:**
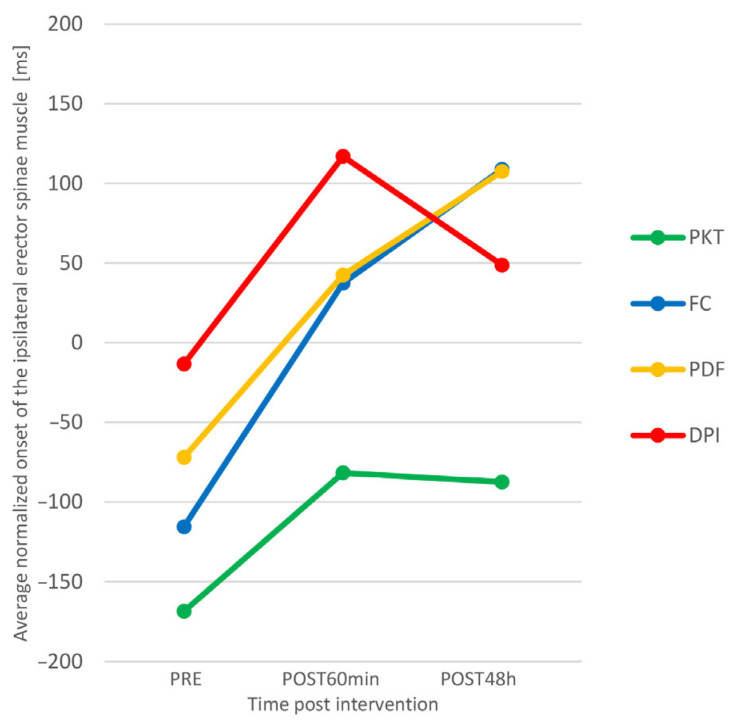
Average normalized onset of the ipsilateral erector spinae muscle (ANONSET_ESI_) interaction plot for 4 groups (PKT—Placebo Kinesio Taping; FC—Functional Correction; PDF—proximal to distal = facilitation; DPI—distal to proximal = inhibition).

Between the three different kinesio taping techniques, we found similarities in the effect of functional correction and kinesio taping muscle facilitation technique (PDF). The trend is the same (see Figure 4 and Figure 5), although, for the FC group, a statistically significant increase in ONSET_ESC_ occurred between all time-points, and for the PDF group, in the second and third measurement points, in relation to the initial one. Since we have not found statistically significant differences in the effects of these two kinesio taping techniques, for extensive practical application, we recommend the use of the muscle facilitation technique because it provides a similar effect of changing neuromuscular function, it is less invasive, and the application procedure is simpler, with less possibility of making mistakes in the preparation and positioning of the patient and application of the material. Our findings could be clinically relevant because kinesio taping is a widespread method often used by many practitioners in the prevention and rehabilitation of various disorders of the neuro-musculoskeletal system. Practical implications can be found in the context of primary prevention through the reduction of motor activation deficits. This is supported by the findings of Schuermans et al. [45] that, in athletes whose contralateral trunk extensors are activated before the hamstring muscles when performing prone hip extension test, there is an eight times higher probability of injury.

## 5. Conclusions

After the application of kinesio taping, there were significant acute and prolonged positive changes in the coordination of muscle activity of the lumbo–pelvic–hip complex. By placing the kinesio tape on the hip flexors through the PDF and DPI principle, a significant increase in ONSET_ESC_ and a decrease in ONSET_ST_ after 60 min and 48 h can occur. Kinesio taping functional correction proved to be the most effective intervention technique, as the ONSET_ESC_ differences between all time-points were statistically significant. By applying the method of neutralizing the recoil effect, we believe that we have improved the concept of preparing and applying placebo kinesio taping, with the potential to become the standard in placebo-controlled research on kinesio taping method effectiveness.

## Figures and Tables

**Table 1:** Descriptive parameters of average normalized onset times of the contralateral erector spinae muscle.

		Time-Point					
		Baseline		POST60min		POST48h	
Group	*n*	Mean	SD	Mean	SD	Mean	SD
**PKT**	14	−10.2	(267.8)	52.7	(231.2)	65.9	(243.7)
**FC**	14	−12.5	(199.1)	103.4	(194.7)	192.7	(264.1)
**PDF**	14	1.3	(140.7)	141.4	(133.1)	183.7	(114.8)
**DPI**	14	−16.6	(197.5)	140.8	(193.6)	147.4	(156.8)

Legend: POST60min—60 min post-intervention; POST48h—48 h post-intervention; SD—Standard Deviation; PKT—Placebo Kinesio Taping; FC—Functional Correction; PDF—proximal to distal = facilitation; DPI—distal to proximal = inhibition.

**Table 2:** Descriptive parameters of average normalized onset times of the ipsilateral erector spinae muscle.

		Time-Point					
		Baseline		POST60min		POST48h	
Group	*n*	Mean	SD	Mean	SD	Mean	SD
**PKT**	14	−168.5	(262.2)	−81.7	(253.5)	−87.4	(264.7)
**FC**	14	−115.5	(215.1)	37.3	(141.5)	108.8	(144.2)
**PDF**	14	−72	(208.2)	42.4	(184.2)	107.4	(158.5)
**DPI**	14	−13.3	(254)	116.9	(226.5)	48.6	(179.1)

Legend: POST60min—60 min post-intervention; POST48h—48 h post-intervention; SD—Standard Deviation; PKT—Placebo Kinesio Taping; FC—Functional Correction; PDF—proximal to distal = facilitation; DPI—distal to proximal = inhibition.

**Table 3:** Descriptive parameters of average time span.

		Time-Point					
		Baseline		POST60min		POST48h	
Group	*n*	Mean	SD	Mean	SD	Mean	SD
**PKT**	14	439.1	(123.7)	410.7	(116.6)	419.7	(160.7)
**FC**	14	411.2	(136.6)	359.9	(140.6)	397.1	(219.2)
**PDF**	14	366.3	(111.3)	365.9	(96.3)	336.9	(82.7)
**DPI**	14	399.4	(65.8)	395.4	(115.5)	376.1	(117.3)

Legend: POST60min—60 min post-intervention; POST48h—48 h post-intervention; SD—Standard Deviation; PKT—Placebo Kinesio Taping; FC—Functional Correction; PDF—proximal to distal = facilitation; DPI—distal to proximal = inhibition.

**Table 4:** Main results of mixed-model ANOVA.

	Group Effect	Time Effect	Interaction
ANONSET_ESC_	F = 0.341	F = 22.152	F = 2.002
	*p* = 0.887	***p* < 0.001**	***p* = 0.048**
		η_p_^2^ = 0.221 **	η_p_^2^ = 0.114 *
ANONSET_ESI_	F = 0.194	F = 17.712	F = 1.689
	*p* = 0.198	***p* < 0.001**	*p* = 0.088
		η_p_^2^ = 0.185 **	
ATS	F = 0.58	F = 0.638	F = 1.571
	*p* = 0.715	*p* = 0.51	*p* = 0.131

Legend: ANONSET_ESC_—average normalized onset of the contralateral erector spinae muscle; ANONSET_ESI_—average normalized onset of the ipsilateral erector spinae muscle; ATS—average time span; *—medium effect; **—large effect.

## Data Availability

Not applicable.
